# Primary Mast Cell Sarcoma of the Maxillary Sinus and Gingiva Mimicking Malignant Neuroendocrine Tumor: A Case Report

**DOI:** 10.1007/s12105-024-01702-w

**Published:** 2024-10-15

**Authors:** Tzu-Chien Cheng, Jim-Ray Chen, Ren-Ching Wang, Kung-Chao Chang, Jen-Fan Hang

**Affiliations:** 1 Division of Pathology, Keelung Chang Gung Memorial Hospital, Keelung, Taiwan; 2 Department of Pathology, China Medical University Hospital, Taichung, Taiwan; 3 Department of Pathology, College of Medicine, National Cheng Kung University Hospital, National Cheng Kung University, Tainan, Taiwan; 4 Department of Pathology and Laboratory Medicine, Taipei Veterans General Hospital, No. 201, Sec. 2, Shipai Rd, Taipei City, 112201 Taiwan; 5 Department of Pathology, School of Medicine, National Yang Ming Chiao Tung University, Taipei, Taiwan; 6 Institute of Clinical Medicine, National Yang Ming Chiao Tung University, Taipei, Taiwan

**Keywords:** Mast cell sarcoma, Mast cell leukemia, Maxillary sinus, Gingiva, Neuroendocrine

## Abstract

Mast cell sarcoma (MCS) is an extremely rare and aggressive malignancy primarily affecting bones, with limited literature associating it with neuroendocrine marker expression. This report presents a rare case of MCS arising in the maxillary sinus and gingiva. A 74-year-old man presented with a progressively enlarging ulcer on the right-sided upper gingiva. Magnetic resonance imaging revealed a 3.4 cm tumor on the floor of the right maxillary sinus. The patient underwent an inferior maxillectomy and right-sided neck dissection. Microscopically, the tumor consisted of monotonous round cells with oval nuclei, vesicular chromatin, inconspicuous nucleoli, and brisk mitoses. A panel of immunohistochemical stains was initially applied to exclude common sinonasal undifferentiated neoplasms, such as sinonasal undifferentiated carcinoma, melanoma, rhabdomyosarcoma, Ewing sarcoma, and lymphoma. The tumor cells showed patchy staining for INSM1 and synaptophysin, but were negative for AE1/AE3, CAM5.2, p40, chromogranin, S100, HMB45, NKX2.2, desmin, CD45 (LCA), CD3, and CD20, with intact INI1 and BRG1 expression. No specific diagnosis could be rendered based on the staining results, leading to consideration of other rare malignancies. Additional staining revealed positivity for CD117, mast cell tryptase, CD13, CD33, CD43, and CD68, confirming the MCS diagnosis. Molecular testing for *KIT* mutation was negative. Subsequent bone marrow biopsy demonstrated infiltration of atypical mast cells, which led to a diagnosis of mast cell leukemia. Despite high-dose chemotherapy, the patient died three months after the initial diagnosis. The undifferentiated epithelioid morphology and unusual aberrant neuroendocrine marker expression posed significant diagnostic challenges. The major differential diagnoses were discussed in this report.

## Introduction

Mast cell sarcoma (MCS) is an extremely rare and highly aggressive neoplasm characterized by the localized proliferation of neoplastic mast cells [1; 2]. Although initially localized, it often disseminates within a few months, leading to a terminal phase of mast cell leukemia. This malignancy is associated with a poor prognosis and limited treatment options [1–3]. A recent report documented 10 new cases of MCS, along with 24 previously reported cases [1]. The combined median age of these cases was 39 years, with nearly equal representation of men and women. Notably, approximately 60% of cases involved bone, while about 20% affected the gastrointestinal tract [1].

Histologically, the neoplastic cells of MCS have a wide morphologic spectrum and may present as medium- to large-sized pleomorphic, epithelioid, or spindle cells. These cells exhibit irregular, lobulated, round to oval nuclei, fine or coarse chromatin, and abundant pale to lightly eosinophilic cytoplasm [1; 4]. Immunohistochemically, the tumor cells commonly express CD117, mast cell tryptase, CD13, CD33, CD43, and CD68. Neoplastic mast cells may also show aberrant expression of CD2 and CD25 [1; 4].

To the best of our knowledge, there is only one case report of a MCS primarily involving maxillary sinus [5]. The expression of neuroendocrine markers, such as synaptophysin, chromogranin, and INSM1, is exceedingly rare in MCS. In this report, we present the clinical, histologic, and immunophenotypic characteristics of a primary mast cell sarcoma arising in the maxillary sinus and gingiva. This tumor exhibits aberrant expression of neuroendocrine markers, which can deceptively mimic a malignant neuroendocrine tumor of the head and neck.

## Case Report

### Clinical Summary

**Fig. 1:**
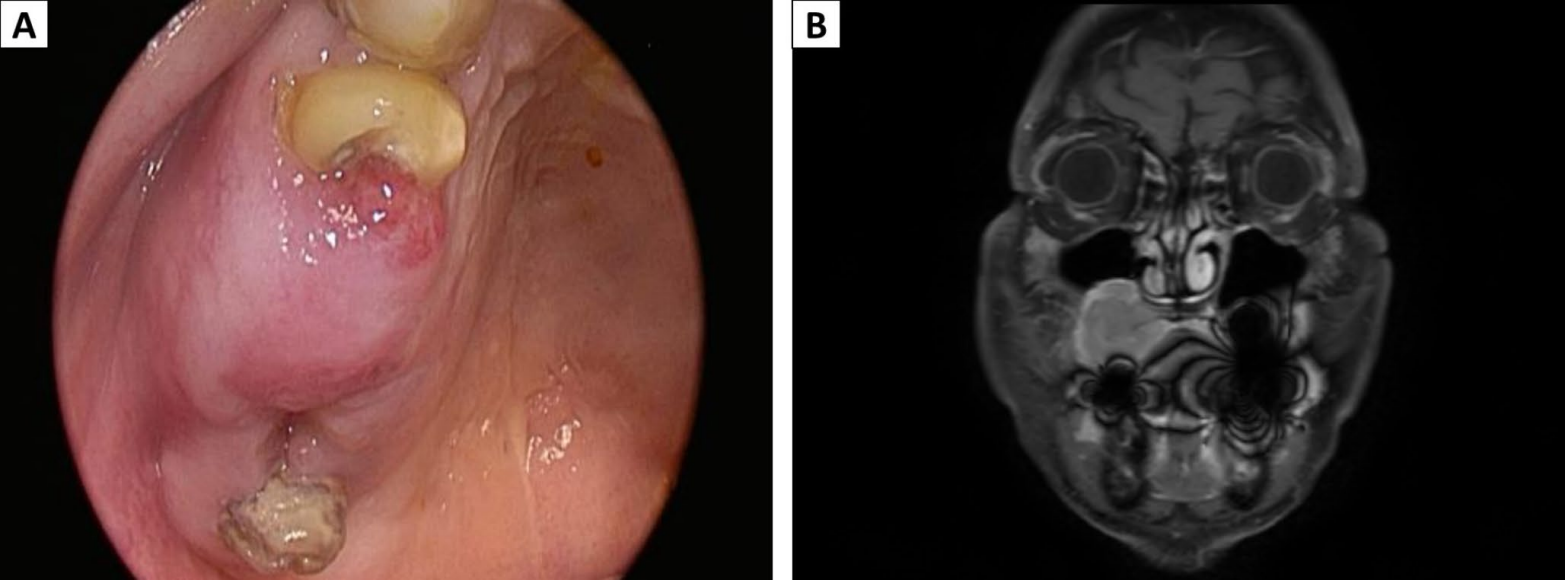
Clinical presentation. (**A**) Intraoral view showing redness and swelling of the gingival mass with ulceration in the posterior portion. (**B**) Coronal magnetic resonance T1-weighted image with contrast enhancement showed a right maxillary mass

The 74-year-old man presented with a progressively enlarging ulcer on the posterior aspect of right-sided upper gingiva and edentulous ridge of maxilla (#15 and #16 area) (Fig. 1A), accompanied with pain for several months. No symptoms of mast cell activation syndrome, such as fever, flushing, or diarrhea, were observed. The patient reported no significant past medical, personal, or family history. On physical examination, no palpable neck lymphadenopathy was noted.

Laboratory tests revealed mild to moderate anemia (Hb = 11.3 g/dL, HCT = 33.2%, MCV = 95.4 fL, and MCH = 32.5 pg) and mild leukopenia (WBC = 3,500 per µL). Blood levels of absolute neutrophil count (ANC) (2251 per µL), eosinophils (1.2% of circulating leukocytes), basophils (0.3% of circulating leukocytes), platelet count (PLT = 186,000 per µL), serum tryptase (3.6 ng/mL, reference range ≤ 11), and lactate dehydrogenase (LDH) (172 U/L, reference range ≤ 250) were normal.

The magnetic resonance imaging (Fig. 1B) of the head and neck region, performed after the administration of intravenous contrast material, revealed a tumor measuring up to 3.4 cm in diameter on the floor of the right maxillary sinus, with associated bone destruction. Subsequent positron emission tomography identified a tumor in the right upper gum (standardized uptake value [SUV] of 5.7, score 4) and a right paratracheal nodal lesion (score 2). Abdominal ultrasonography showed no hepatomegaly or splenomegaly.

### Pathological Findings

**Fig. 2:**
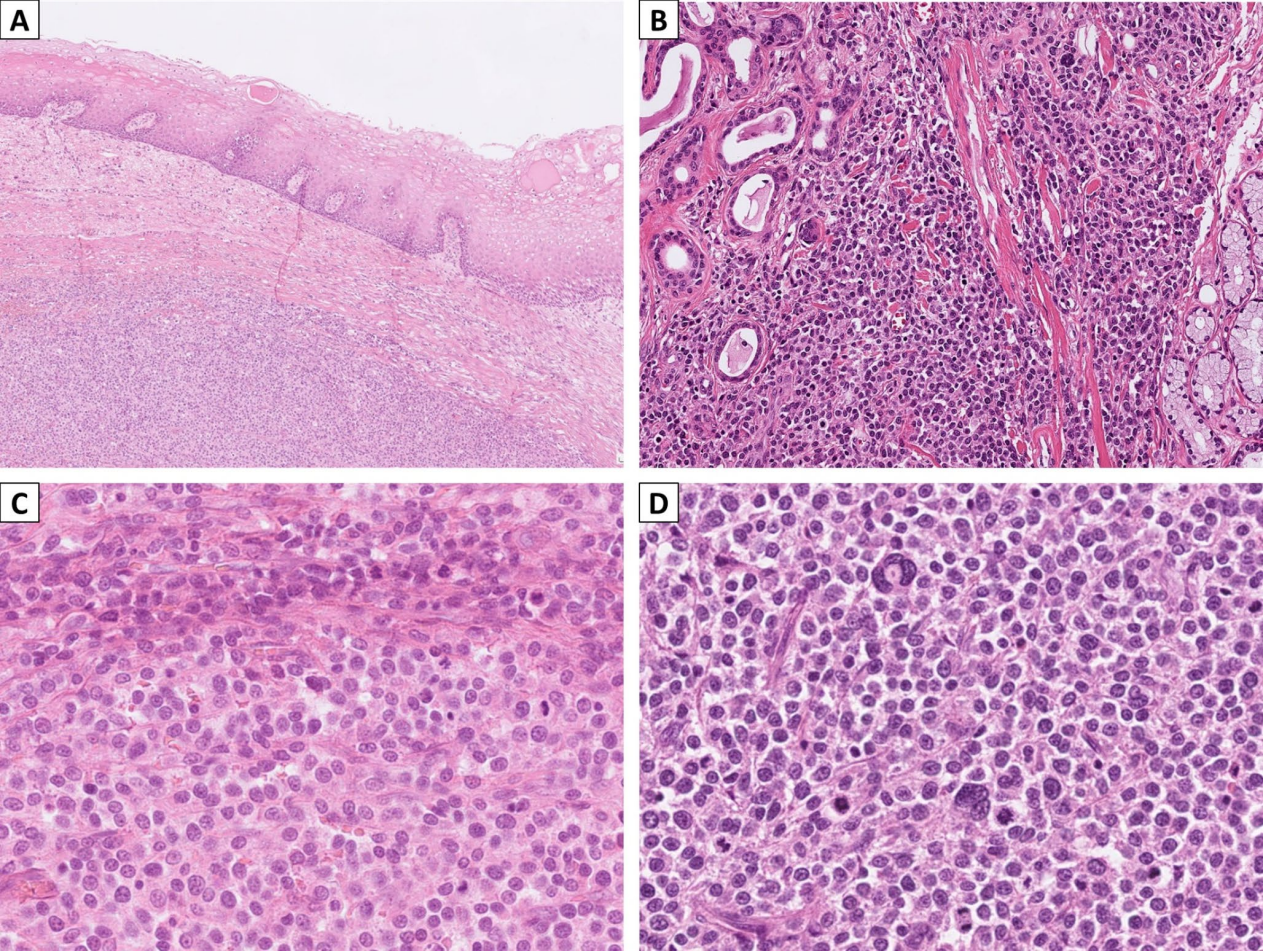
Histopathologic features of mast cell sarcoma affecting the gingiva and the maxillary sinus. (**A**) A low-power photomicrograph depicted the subepithelial tumor cells and the overlying stratified squamous epithelium (H&E, 100X). (**B**) A medium-power photomicrograph showed the infiltrating neoplastic cells and the atrophic minor salivary glands (H&E, 200X). (**C**) A high-power photomicrograph revealed a proliferation of monomorphic tumor cells with oval nuclei, indistinct to punctate nucleoli, eosinophilic-granular to pale cytoplasm, and brisk mitoses (H&E, 400X). (**D**) Rare multinucleated giant tumor cells were also observed (H&E, 400X)

A wide excision (inferior maxillectomy) and right-sided neck dissection (levels I to III) were performed. Gross examination of the excised tissue revealed an infiltrative white tumor measuring 2.8 × 2.5 × 2.2 cm, invading the maxilla. Histologic examination showed a subepithelial tumor composed of monotonous medium-sized round cells with oval nuclei, vesicular to finely granular chromatin, inconspicuous to punctate nucleoli, and ample eosinophilic to pale cytoplasm, arranged in diffuse solid sheets with an intervening vascular network (Fig. 2A and C). Mild to moderate pleomorphism was observed, along with rare multinucleated giant cells (Fig. 2D). No spindle cells or eosinophil aggregation were present. Frequent mitoses were noted (> 10/10 high-power fields). The tumor infiltrated the stroma, blood vessels, nerve bundles, trabecular bone, and atrophic minor salivary glands without apparent desmoplastic reaction (Fig. 2B). The overlying squamous epithelium showed no dysplasia or tumor involvement. All dissected lymph nodes were free of malignancy.

**Fig. 3:**
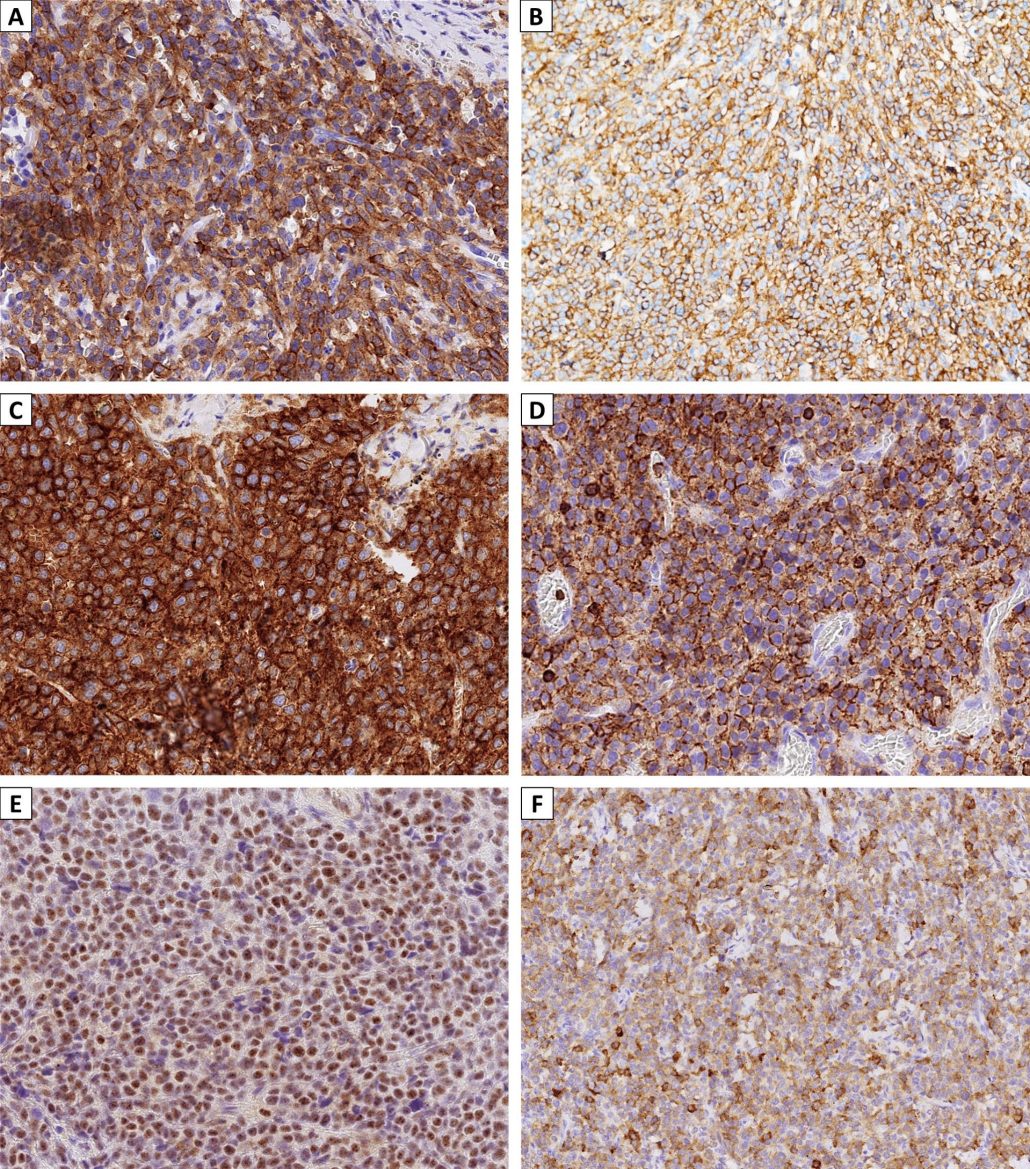
Immunohistochemical stains showed that the neoplastic mast cells expressed (**A**) CD117, (**B**) mast cell tryptase, (**C**) CD33, (**D**) CD43, (**E**) INSM1, and (**F**) synaptophysin (all in 400X)

A panel of immunohistochemical stains was initially performed. Tumor cells were negative for epithelial markers AE1/AE3 and CAM5.2, and also negative for p40, NUT, chromogranin, desmin, NKX2.2, S100, SOX10, HMB45, GATA3, CD45 (LCA), CD3, and CD20, with intact INI1 and BRG1 nuclear expression. They demonstrated patchy, moderate to strong positivity for INSM1 and synaptophysin (Fig. 3E-F).

While excluding most common sinonasal undifferentiated neoplasms, the H&E morphology suggested consideration of hematologic neoplasms despite negative expression for CD45 (LCA), CD3, and CD20. Additional immunohistochemical stains were performed. The tumor cells were negative for CD25, myeloperoxidase (MPO), CD34, CD123, CD138, and CD163 but strongly positive for CD117, CD13, and CD33 (Fig. 3A and C) and focally positive for mast cell tryptase, CD30, CD43, and CD68 (Fig. 3B and D). *KIT* mutation analysis was negative for the p.D816V mutation. These histomorphologic and immunophenotypic findings were consistent with those of a mast cell sarcoma.

**Fig. 4:**
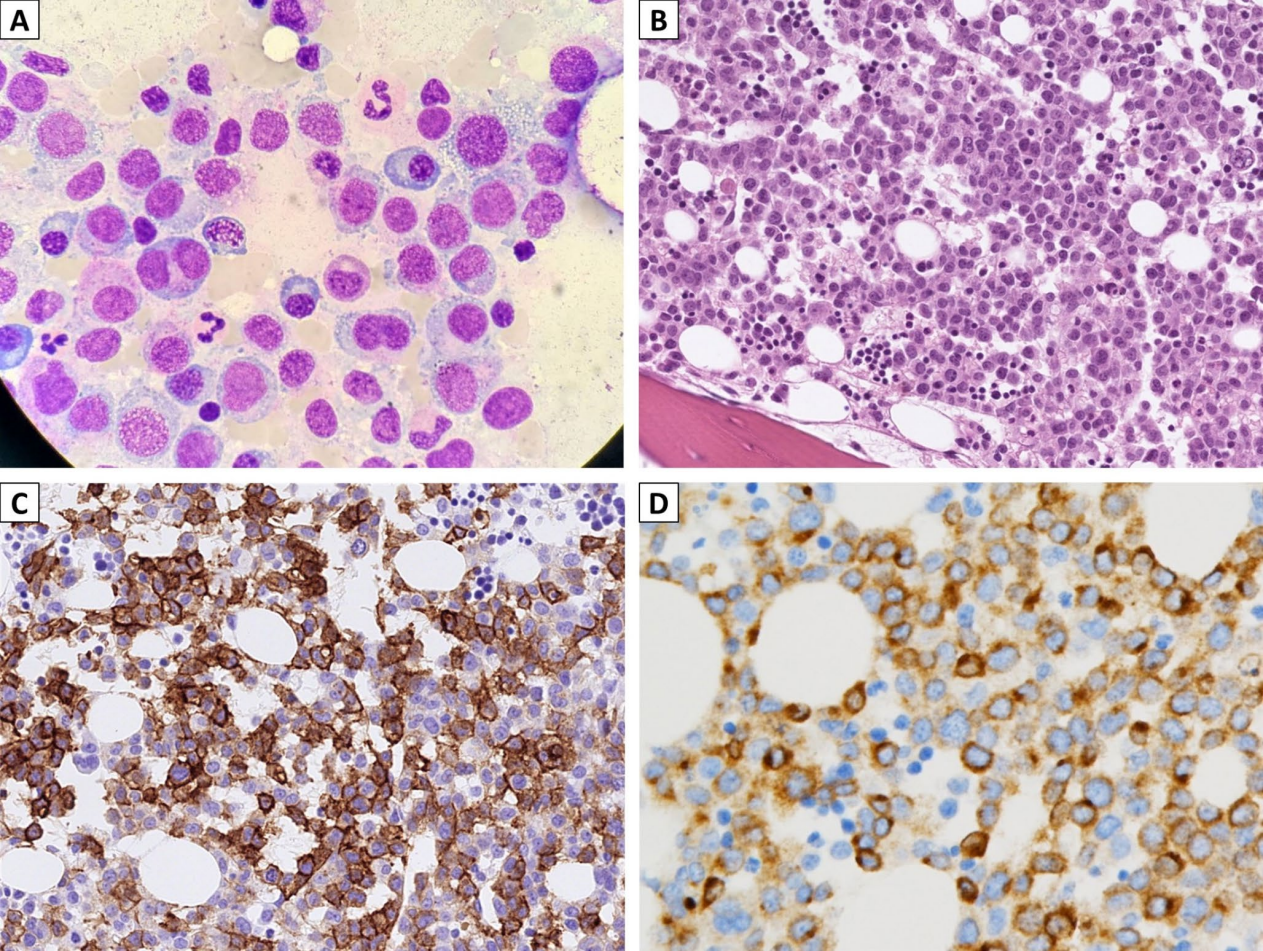
The subsequent bone marrow aspirate smear (**A**) and trephine biopsy (**B**) revealed dense infiltrates composed of more than 20% hypogranular atypical mast cells, type II, which were positive for CD117 (**C**) and mast cell tryptase (**D**), consistent with mast cell leukemia (**A**, Liu stain, 1000X; **B**, H&E, 400X; **C-D**, 400X)

Subsequent bone marrow aspirate, trephine biopsy, and flow cytometry study were performed after the pathologic diagnosis. The bone marrow aspirate smear (Fig. 4A) showed numerous immature mast cells, accounting for 70% of total marrow elements, with variably sized nuclei, occasionally lobulated (type II hypogranular atypical mast cells), an increased nuclear-cytoplasmic ratio, and basophilic granules in the cytoplasm. The bone marrow biopsy (Fig. 4B) revealed sheets of neoplastic cells occupying over 60% of marrow space, characterized by oval nuclei, moderate eosinophilic to pale cytoplasm, and frequent mitoses. Immunohistochemistry revealed positivity for CD117, mast cell tryptase (Fig. 4C and D), and CD30, and negativity for CD2, CD25, CD34, and MPO. Flow cytometry of the bone marrow confirmed expression of CD117, CD13, and CD33. These findings supported the diagnosis of mast cell leukemia.

### Follow-up Information

Low-dose chemotherapy with vincristine and methotrexate was administered. However, pancytopenia progressed and jaundice (direct bilirubin = 8.9 mg/dL) developed 2 months after diagnosis. Magnetic resonance cholangiopancreatography revealed newly formed multiple liver nodules and a splenic nodule, suggesting possible disease progression. The treatment was shifted to high-dose chemotherapy with cytarabine. Unfortunately, the patient experienced respiratory failure and died three months after the initial diagnosis.

## Discussion

Primary MCS involving paranasal sinus and head and neck region is exceedingly rare. A comprehensive review of the literature revealed only one previously documented case in the maxillary sinus, although the title of that report incorrectly referring it as “mast cell carcinoma” [5]. Other reported primary sites of MCS in the head and neck region include scalp, temporal and parietal bones, eye, ear, and inner lip [4].

Histologically, the tumor was characterized by monotonous epithelioid cells with oval nuclei, punctate nucleoli, discernible cytoplasm, and brisk mitotic activity. An epithelial malignancy should be considered as the primary differential diagnosis. Emerging and newly classified sinonasal malignancies present substantial diagnostic challenges. Various algorithms have been proposed to guide the differential diagnostic work-up of poorly differentiated or undifferentiated sinonasal small round cell tumors [6; 7]. These algorithms integrate ancillary testing to classify these tumors as either epithelial neoplasms or non-epithelial malignancies. In our case, a comprehensive immunohistochemical panel, as recommended by these algorithms, was employed. The absence of AE1/AE3, CAM5.2, p40, and NUT effectively ruled out the majority of sinonasal carcinomas. Additionally, negative staining for NKX2.2, CD99, desmin, S100, SOX10, HMB45, GATA3, CD45 (LCA), CD3, and CD20 further excluded diagnoses of Ewing sarcoma, rhabdomyosarcoma, melanoma, and the majority of non-Hodgkin lymphomas.

Patchy staining for neuroendocrine markers such as INSM1 and synaptophysin can present a diagnostic pitfall, particularly when a tumor exhibits a monotonous epithelioid morphology with vesicular chromatin. The absence of cytokeratin expression typically excludes neuroendocrine tumors or neuroendocrine carcinomas from the differential diagnosis. Additionally, the maxillary location and the negative staining for S100, SOX10, and GATA3 do not support a diagnosis of paraganglioma or olfactory neuroblastoma. According to a recent review of head and neck neoplasms [8], several non-neuroendocrine tumors may exhibit neuroendocrine marker expression, including sinonasal undifferentiated carcinoma, Ewing family tumors, and SWI/SNF complex-deficient sinonasal carcinoma. Therefore, careful interpretation and exclusion of other neuroendocrine mimickers are crucial when diagnosing a poorly differentiated or undifferentiated head and neck neoplasm. There is no previous documentation in the literature associating MCS with aberrant expression of neuroendocrine markers [9].

In the current case, the unusual staining results and unique infiltration pattern, where the tumor did not induce a desmoplastic response, prompted additional consideration of CD3- and CD20-negative hematologic malignancies, such as plasma cell neoplasm, myeloid sarcoma, blastic plasmacytoid dendritic cell neoplasm, mast cell sarcoma, and anaplastic large cell lymphoma. Finally, the positivity of CD117, mast cell tryptase, and other mast cell markers confirmed the diagnosis. The prognosis of MCS is dismal, with a median survival of 24 months [1]. In this case, the tumor rapidly progressed to mast cell leukemia, and the patient passed away only 3 months after diagnosis.

There was a discrepancy in CD30 expression between the primary mast cell sarcoma in the maxillary sinus and the bone marrow mast cells. In the maxillary sinus, most tumor cells (over 95%) were negative for CD30, with only a few hotspots showing positivity. In contrast, the majority of neoplastic mast cells in the bone marrow were CD30-positive. Variable expression of CD30 in mast cell sarcoma has been reported in previous studies [1; 4; 10]. However, discordance in CD30 expression between different organs in mast cell sarcoma has not been well-documented in the literature. Previous studies have shown that CD30 is predominantly expressed in advanced mast cell neoplasms, suggesting that neoplastic mast cells may enhance tumor growth and survival through CD30-related NF-κB signaling and autocrine interactions between CD30 and its ligands [11]. The bone marrow specimen was obtained two months after the primary tumor, which may explain the observed CD30 expression in the bone marrow. However, it is important to note that the association of CD30 expression with aggressive systemic forms of mastocytosis has not been consistently observed in more recent research [12; 13]. Further studies are needed to investigate the pathogenesis.

In conclusion, we described the clinicopathologic and immunophenotypic characteristics of a rare and challenging case of MCS affecting the maxillary sinus and gingiva. This tumor showed unusual aberrant expression of neuroendocrine markers, which could falsely suggest a malignant neuroendocrine tumor in the head and neck region. Awareness of the morphologic features and a comprehensive ancillary work-up are essential for the accurate diagnosis of this uncommon condition.

## Data Availability

The data that support the findings of this study are available upon reasonable request.
